# Acceptance of Artificial Intelligence in Clinical Practice Among Chinese Physicians: Nationwide Cross-Sectional Survey Using Extended Unified Theory of Acceptance and Use of Technology and Explainable Machine Learning

**DOI:** 10.2196/85270

**Published:** 2026-04-16

**Authors:** Xuefei Shi, Zhanxiao Tian, Qi Guo, Boxuan Qiao, Xiaolong Wang

**Affiliations:** 1 Department of Cardiology, Shuguang Hospital, Shanghai University of Traditional Chinese Medicine, 185 Pu'an Road, Huangpu District, Shanghai, 200021, China, 86 13501991450; 2 Research and Development Center, KINGYEE (Beijing) Co., Ltd., Beijing, China; 3 College of Computer Science, Zhejiang University, Hangzhou, China

**Keywords:** machine learning, physicians, attitude of health personnel, UTAUT, Unified Theory of Acceptance and Use of Technology, SHAP, Shapley Additive Explanations

## Abstract

**Background:**

Artificial intelligence (AI) is rapidly transforming clinical practice, yet empirical evidence on Chinese physicians’ acceptance of AI medical tools remains scarce at the national level.

**Objective:**

This study aimed to evaluate the current acceptance of AI medical tools among Chinese physicians, identify key determinants, and elucidate underlying mechanisms using an extended Unified Theory of Acceptance and Use of Technology (UTAUT) and explainable machine learning.

**Methods:**

A nationwide cross-sectional survey was conducted from January to April 2024, recruiting 4024 in-service physicians across 29 provincial-level administrative units in China via stratified random sampling. The questionnaire incorporated 5 UTAUT constructs—performance expectancy, effort expectancy, social influence, facilitating conditions (FC), and a newly introduced “positive impact” dimension. Psychometric properties were validated through exploratory and confirmatory factor analyses. Structural equation modeling assessed direct and moderated effects, with hospital level, professional title, AI familiarity, and future optimism as moderators. Six classification models were compared for predictive performance; balanced random forest was selected, and model interpretability was evaluated using Shapley Additive Explanations (SHAP).

**Results:**

Overall acceptance exceeded 90% across subgroups. Structural equation modeling showed that performance expectancy, social influence, FC, and positive impact significantly and positively predicted physicians’ behavioral intention to use AI medical tools. Six negative moderation effects were identified. The random forest achieved 85.6% accuracy and an area under the receiver operating characteristic curve of 0.836; SHAP analysis identified organizational support (FC_HospPromoteAI) as the feature with the highest mean absolute SHAP value, though all effect sizes were modest.

**Conclusions:**

Chinese physicians exhibit high acceptance of AI medical tools, mainly driven by organizational support and perceived clinical benefits. The combined use of extended UTAUT and explainable AI provides actionable insights for targeted AI implementation strategies in health care.

## Introduction

Artificial intelligence (AI) is transforming health care delivery worldwide by enhancing efficiency and quality through automation and intelligent systems [1,2]. Its applications include medical imaging analysis, clinical decision support, and chronic disease management. Convolutional neural networks based on deep learning have demonstrated superior performance in imaging diagnostics [3]. AI medical tools can reduce diagnostic time by approximately 30% and improve accuracy by about 10%, particularly in oncology and cardiovascular medicine [4]. In recent years, large language models such as ChatGPT, Med-PaLM (Google LLC), and domestic systems like DeepSeek and Doubao (ByteDance Ltd.) have shown significant potential in generating medical records, translating scientific literature, supporting clinical decision-making, and facilitating physician-patient communication [5]. In China, the adoption of AI tools for imaging analysis and decision support has steadily increased, especially in tertiary hospitals [6]. However, concerns about algorithmic bias, data privacy, and accountability remain key barriers to widespread adoption, making physicians’ acceptance a critical factor in successful implementation [7,8].

Globally, physicians’ attitudes toward AI medical tools are complex. In Western countries, about half of physicians recognize the potential of AI to improve diagnostic efficiency, but many express concerns about privacy and ethics [9,10]. In the United Kingdom, some physicians have refused to use AI imaging tools due to insufficient training [11]. Algorithmic bias, such as racially skewed diagnostic errors, has been shown to undermine trust, particularly when AI-generated recommendations come from large language models [12]. A small-scale Japanese survey indicated that high learning costs also discourage adoption of AI medical tools [13]. Health system differences further influence acceptance: physicians in predominantly private health care systems in Western countries generally enjoy higher autonomy, more resources, and shorter working hours (about 40 h/wk) [14], whereas in China’s predominantly public health care system, physicians often work more than 50 hours/week [15]. Existing Chinese studies are mostly regional or small-scale, lack nationally representative data, and rarely integrate explainable machine learning approaches [5,16].

The technology acceptance model identifies perceived usefulness and perceived ease of use as core drivers of adoption [17]. The Unified Theory of Acceptance and Use of Technology (UTAUT) expands this framework by adding social influence (SI), facilitating conditions (FC), and risk perceptions [18]. However, most UTAUT-based studies are conducted in Western health care contexts and have not addressed the unique challenges of China’s public health care system, such as high workloads and reduced autonomy [15]. Furthermore, the positive impacts (PIs) of AI—such as improving efficiency, enhancing diagnostic accuracy, and strengthening physician-patient relationships—have not been systematically incorporated into the UTAUT framework.

Therefore, this study aimed to assess the current acceptance of AI medical tools among Chinese physicians, identify key influencing factors and potential moderating effects, and apply explainable machine learning to uncover the complex mechanisms underlying physicians’ behavioral intentions (BIs) toward AI adoption.

## Methods

### Study Design and Participants

This study used a nationwide cross-sectional survey (January-April 2024) to assess Chinese physicians’ acceptance of AI medical tools and related determinants. The questionnaire was developed by adapting established scales from the UTAUT [17]. It included 4 original constructs—performance expectancy (PE), effort expectancy (EE), SI, and FC—and an additional “PI” construct. The PI items were derived from the concept of “perceived usefulness” [18] and contextualized to capture specific perceived clinical benefits. The conceptual distinction is clarified as follows: PE items focused on task-level efficiency gains (eg, “AI medical tools can improve health care efficiency”), whereas PI items captured broader clinical and patient-oriented outcomes (eg, “AI can help improve patients’ clinical outcomes”; “AI can improve diagnostic accuracy”). While PE reflects expectations about operational performance improvements, PI emphasizes the perceived benefits for clinical quality and patient welfare.

**Figure 1.**
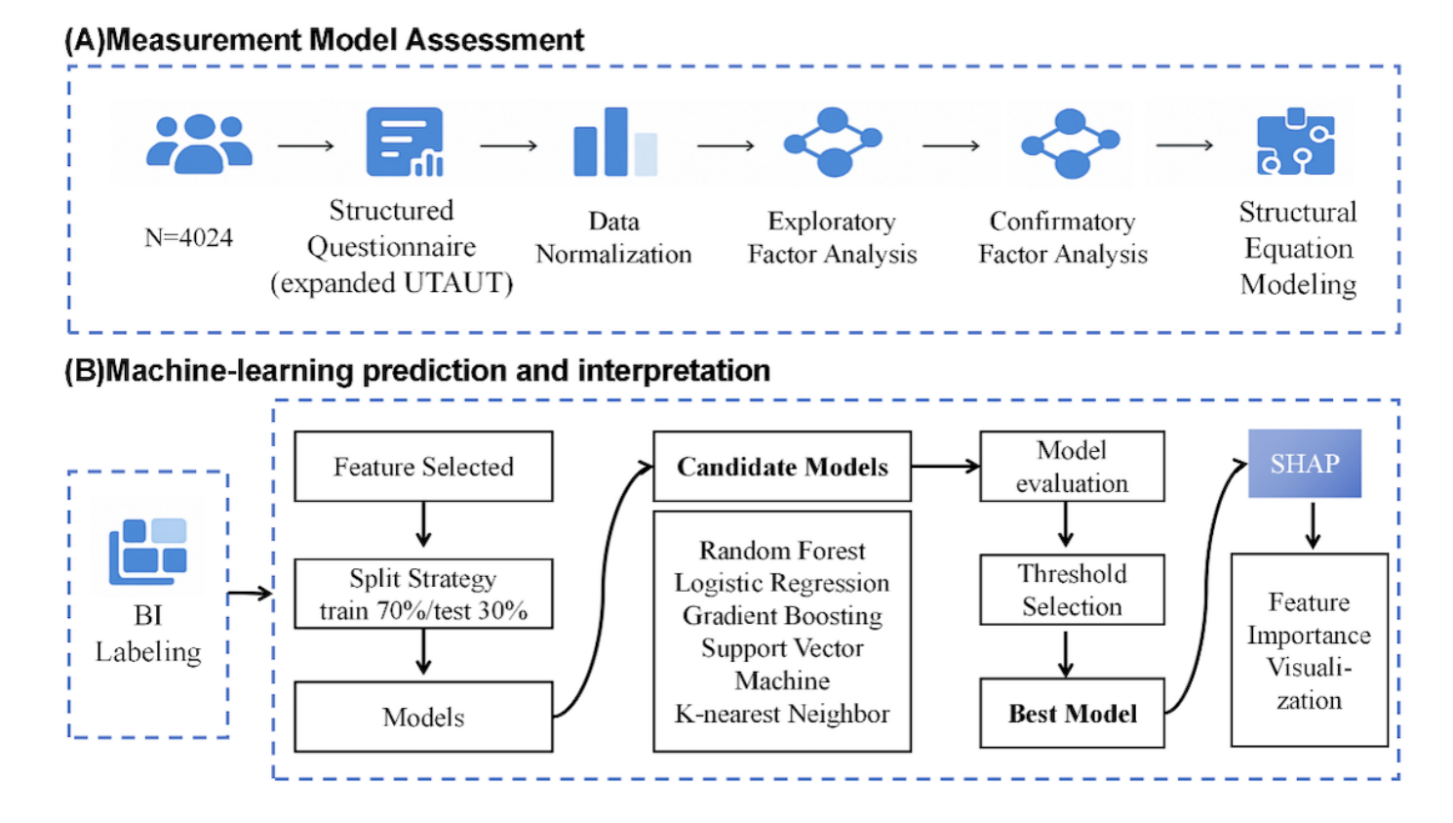
Overview of study design and analysis workflow. (A) Psychometric and structural modeling. (B) Machine learning prediction and interpretation. BI: behavioral intention; SHAP: Shapley Additive Explanations; UTAUT: Unified Theory of Acceptance and Use of Technology.

Structured questionnaires were distributed online via Medlive, which is the largest leading professional academic platform for physicians in China, possessing a comprehensive database of verified real-name physicians. To ensure representativeness, we used a stratified random sampling strategy based on 2 key dimensions: geographic location (31 provincial-level administrative regions in mainland China) and hospital classification (tertiary, secondary, and primary institutions). The sampling aimed to achieve nationwide coverage across all provinces rather than predetermined numerical quotas for each stratum. The platform randomly pushed survey invitations to eligible physicians within these strata. A total of 4725 physicians clicked the survey invitation link, of whom 4030 (85.3%) completed all required items. After excluding 6 responses with inconsistent data patterns, 4024 valid questionnaires were retained for analysis, ultimately covering 29 of 31 provincial-level regions. The overall study framework is illustrated in Figure 1.

### Measurement Model Assessment

The instrument comprised 32 items across 6 dimensions: BI, PE, EE, SI, FC, and PI. BI was assessed using 6 dichotomous items (1=willing, 0=unwilling). PE, PI, SI, and FC each contained 4 items scored on a 4-point Likert scale (1=strongly disagree to 4=strongly agree). All construct scores were linearly transformed to a 0-100 scale for comparability across dimensions.

Exploratory factor analysis was conducted using principal component analysis with Varimax rotation; factors with eigenvalues greater than 1 were retained. Sampling adequacy and sphericity were evaluated using the Kaiser-Meyer-Olkin statistic and Bartlett’s test, respectively [16]. Confirmatory factor analysis was then performed within a structural equation modeling (SEM) framework, and model fit was evaluated using root mean square error of approximation (RMSEA), comparative fit index (CFI), and Tucker-Lewis index (TLI) according to established cutoffs [17]. Internal consistency (Cronbach α), composite reliability (CR), and average variance extracted (AVE) were computed for each construct.

BI was measured using 6 dichotomous (yes or no) items. We acknowledged that weighted least squares mean and variance-adjusted or robust diagonally weighted least squares estimators would be more appropriate for categorical indicators. However, given that the primary analysis used maximum likelihood (ML) estimation consistently across all constructs, we reported the ML results while noting this methodological consideration.

SEM estimated the direct effects of PE, EE, SI, FC, and PI on BI while controlling for hospital level and professional title [18]. Moderation effects were examined for hospital level, AI familiarity, and future optimism by standardizing variables, creating product-term interactions, and entering them hierarchically into regression models [19]. Multicollinearity was evaluated using variance inflation factors.

### Machine Learning Prediction and Interpretability

To capture potential nonlinear and high-dimensional associations, 6 supervised classifiers were compared: logistic regression (LR), support vector machine, random forest (RF), gradient boosting (GB), extreme gradient boosting, and *k*-nearest neighbors [20-25]. The binary outcome was defined as high acceptance (BI score ≥ 83.3, corresponding to “yes” responses on 5 or more of the 6 items; n=3622, 90%) versus moderate or low acceptance (BI score <83.3; n=402, 10%). Data were partitioned into training (n=2817, 70%) and testing (n=1207, 30%) sets using stratified sampling to preserve class proportions. Five-fold stratified cross-validation on the training set was used for model selection and hyperparameter tuning. For tree-based models, key hyperparameters (max_depth, n_estimators, min_samples_split) were optimized via grid search.

Model performance was evaluated using the area under the receiver operating characteristic curve (AUC), accuracy, sensitivity, specificity, and *F*_1_-score. Given class imbalance, precision-recall AUC was additionally considered for robustness [26]. Decision thresholds were optimized using the Youden index to balance sensitivity and specificity [27].

The balanced RF was selected for Shapley Additive Explanations (SHAP) interpretation for three reasons: (1) it achieved the highest accuracy (85.6%) and sensitivity (87.3%) among all models, which is critical for identifying physicians with potential implementation barriers; (2) tree-based SHAP values provide additive, locally accurate explanations that are well-suited for identifying feature interactions; and (3) the class-balancing mechanism addresses the 9:1 class imbalance, producing more reliable feature importance estimates for the minority class. While LR and GB achieved equivalent or higher AUC (0.840), we prioritized sensitivity and interpretability for this exploratory analysis. SHAP was applied to quantify each feature’s contribution to the predicted probability of high AI acceptance, providing global importance rankings (mean absolute SHAP values) and local interpretability (summary plots) [28].

### Statistical Analysis

Categorical variables were summarized as counts and percentages, and continuous variables were reported as medians with IQRs. Group comparisons used the chi-square test or Fisher exact test for categorical variables and the Wilcoxon rank-sum test for continuous variables, as appropriate. Two-sided *P* values <.05 were considered statistically significant; when multiple pairwise comparisons were performed, Bonferroni correction was applied. All statistical analyses were conducted in Python (version 3.10.0) using standard libraries (pandas, NumPy, SciPy, and statsmodels) for inference and scikit-learn for model-related procedures.

### Ethical Considerations

The study was conducted in accordance with the Declaration of Helsinki. Prior to data collection, all participants were presented with an electronic informed consent statement detailing the study objectives, its voluntary nature, and the privacy measures. Participants were required to confirm their agreement by clicking a mandatory “I Agree” button to proceed to the questionnaire. As the study involved a noninterventional, anonymous survey of professionals, formal institutional review board approval was waived in accordance with the operational guidelines of the data collection platform (Medlive), provided that strict data privacy and anonymity protocols were enforced. All participants provided informed consent prior to data collection. No financial compensation or incentives were provided to participants for their involvement in this study.

## Results

### Sample Characteristics

**Figure 2.**
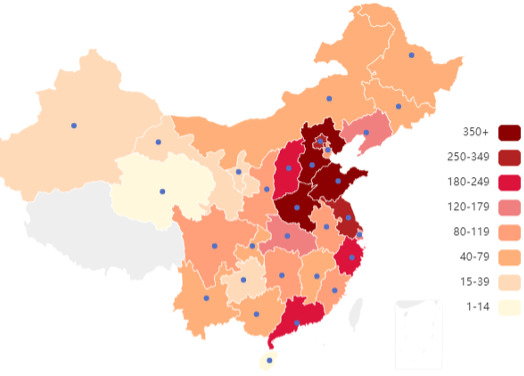
Geographic distribution of physicians across China (n=4024). Provinces are shaded by the number of participants, with darker colors indicating higher counts. A total of 29 provinces were covered in the survey. Blue dots represent provincial capital cities.

A total of 4024 in-service physicians from mainland China were included. As shown in Figure 2, the sample covered 29 provincial-level regions, with most participants located in East China (n=1143, 28.4%) and North China (n=978, 24.3%). Tertiary hospitals accounted for 3114 (77.4%), followed by secondary hospitals (n=717, 17.8%) and primary care institutions (193, 4.8%). Regarding professional title, 2933 (72.9%) held senior titles (chief or associate chief), 1040 (25.8%) held intermediate titles, and 51 (1.3%) were junior or ungraded.

### Measurement Properties of the Questionnaire

The AVE for BI was 0.40, slightly below the conventional threshold of 0.50. This lower AVE is consistent with the strong ceiling effect observed in BI responses, where over 90% (n=3621) of participants indicated willingness to use medical AI across all 6 dimensions (range: 90.6%‐96.4%). Despite the lower AVE, BI demonstrated good internal consistency through CR (0.80) and Cronbach α (0.80), with all standardized factor loadings exceeding 0.59.

As detailed in Table S1 in Multimedia Appendix 1, exploratory factor analysis indicated adequate sampling and factorability (Kaiser-Meyer-Olkin=0.898; Bartlett test *χ*²=47,985.41; *P*<.001). Based on eigenvalues greater than 1 and scree plot inspection, 6 factors were extracted, explaining 62.8% of the total variance, which is consistent with the extended UTAUT specification. All standardized factor loadings exceeded 0.40 (Table S1 in Multimedia Appendix 1), and internal consistency was acceptable to good across constructs (mean Cronbach *α* >0.78).

Confirmatory factor analysis further supported the 6-factor model with good fit (RMSEA=0.043; CFI=0.937; TLI=0.930). CR exceeded 0.80 for all constructs, and AVE was greater than 0.50 for every construct except BI (AVE=0.478), indicating satisfactory convergent validity (Table 1). Discriminant validity was supported, as the square root of each construct’s AVE on the diagonal was greater than the corresponding interconstruct correlations (Table 2).

**Table 1.** Convergent validity of constructs for Chinese physicians’ acceptance of artificial intelligence medical tools. All factor loadings are standardized.

Construct	Cronbach α	CR^a^	AVE^b^	Average loading
EE^c^	0.894	0.915	0.642	0.766
PE^d^	0.818	0.830	0.549	0.729
PI^e^	0.792	0.861	0.608	0.700
SI^f^	0.828	0.851	0.588	0.739
FC^g^	0.852	0.849	0.587	0.774
BI^h^	0.788	0.846	0.478	0.691

a CR: composite reliability.

b AVE: average variance extracted.

c EE: effort expectancy.

d PE: performance expectancy.

e PI: positive impact.

f SI: social influence.

g FC: facilitating conditions.

h BI: behavioral intention.

**Table 2.** Discriminant validity for Chinese physicians’ acceptance of artificial intelligence medical tools.

Construct	EE^a^	PE^b^	PI^c^	SI^d^	FC^e^	BI^f^
EE	*0.766*					
PE	0.321	*0.730*				
PI	−0.063	0.195	*0.702*			
SI	0.203	0.598	0.122	*0.742*		
FC	0.179	0.590	0.202	0.536	*0.778*	
BI	0.118	0.363	0.156	0.327	0.438	*0.633*

a EE: effort expectancy.

b PE: performance expectancy.

c PI: positive impact.

d SI: social influence.

e FC: facilitating conditions.

f BI: behavioral intention.

### Acceptance Across Subgroups

**Figure 3.**
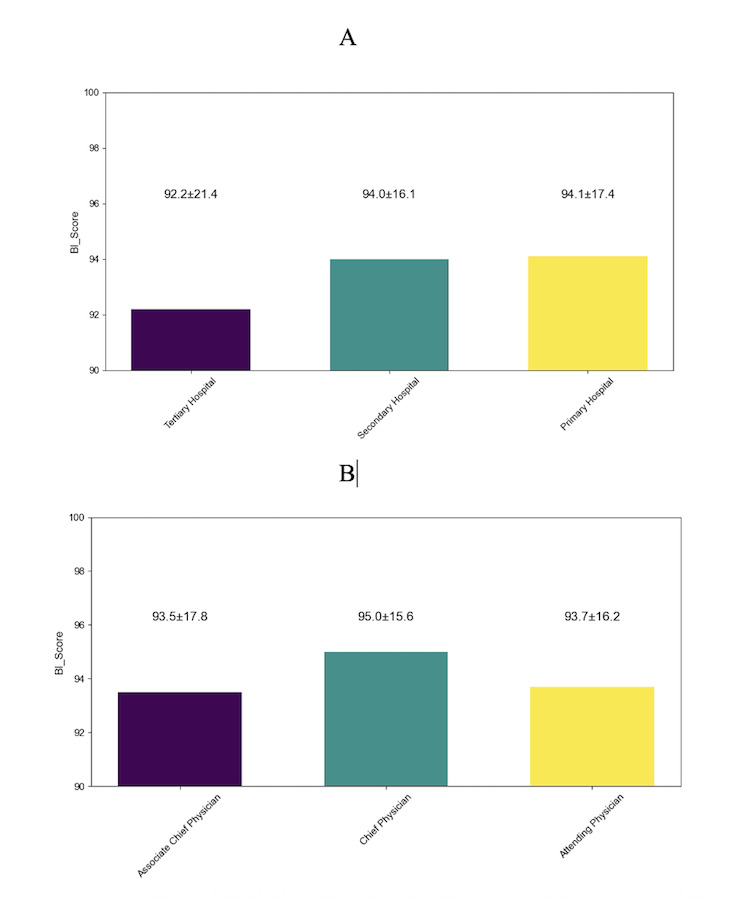
Overall acceptance of artificial intelligence medical tools across subgroups: (A) by hospital level and (B) by professional title. Categories with n<50 were excluded from inferential tests. Bars indicate the proportion reporting willingness (BI=1). BI score was computed as the unweighted arithmetic mean of the 6 items and linearly transformed to a 0‐100 scale. BI: behavioral intention.

As shown in Figure 3, overall willingness to adopt AI medical tools exceeded 90% (3622/4024). Descriptive analysis of the aggregated BI score revealed a negative skewness of −3.55 and a kurtosis of 13.91, statistically confirming the nonnormal distribution associated with the ceiling effect. For inferential analyses, categories with value n less than 50 were excluded (private hospitals, n=29; clinics, village health posts, or outpatient departments, n=11; junior title, n=46; ungraded, n=5). One-way ANOVA indicated no significant between-group differences by hospital level and a borderline difference by professional title that became nonsignificant after Bonferroni correction.

### Structural Model Results

**Figure 4.**
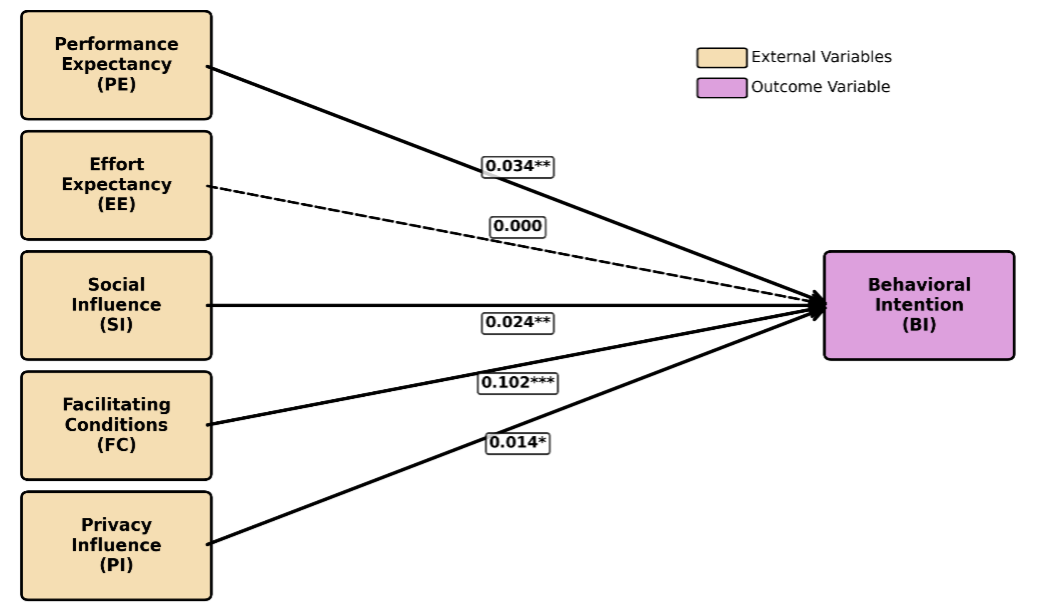
Structural path model based on the extended Unified Theory of Acceptance and Use of Technology framework. Solid lines indicate statistically significant paths, dashed lines indicate nonsignificant paths. Path coefficients are standardized β values, with significance levels denoted as *P*<.05 (*), *P*<.01 (**), and *P*<.001 (***). External variables (performance expectancy, effort expectancy, social influence, facilitating conditions, positive impact) are shown in beige and the outcome variable (BI) is shown in purple (*R*² for BI=0.216). BI: behavioral intention.

The extended UTAUT structural model demonstrated a good fit to the data (RMSEA=0.043; CFI=0.948; TLI=0.941). As shown in Figure 4, PE (*β*=.03, 95% CI 0.014-0.055; *P*=.001), SI (*β*=.02, 95% CI 0.010-0.039; *P*<.001), FC (*β*=.10, 95% CI 0.085-0.119; *P*<.001), and PI (*β*=.01, 95% CI 0.000-0.028; *P*=.047) were positive predictors of BI, whereas EE was not significant (β≈0, 95% CI −0.014 to 0.014]; *P*=.99). The model explained 21.6% of the variance in BI (*R*²=0.216).

### Moderation Analysis

To examine whether background variables altered the primary UTAUT paths, product-term interactions were created using standardized latent variables for the following moderators: hospital level (W5), professional title (W7), AI familiarity (N1), and future optimism (N5). Hierarchical regression identified 6 negative interaction effects (*P* values <.05), indicating attenuation of the corresponding main effects at higher levels of the moderator (Table 3). Variance inflation factors ranged from 1.06 to 1.99 (mean 1.54), suggesting no multicollinearity concerns. Other tested interactions were not statistically significant.

**Table 3.** Moderation effects on Unified Theory of Acceptance and Use of Technology paths (standardized coefficients).

Interaction (moderator × predictor)	β	*P* value	Direction
EE^a^ × W5^b^	−.054	.004	Negative
EE × N1^c^	−.037	.03	Negative
FC^d^ × N1	−.048	.01	Negative
EE × N5^e^	−.043	.01	Negative
FC × N5	−.074	<.001	Negative
PI^f^ × N5	−.054	.001	Negative

a EE: effort expectancy.

b W5: hospital level.

c N1: artificial intelligence familiarity.

d FC: facilitating conditions.

e N5: future optimism.

f PI: positive impact.

### Explainable Prediction Analysis

**Figure 5.**
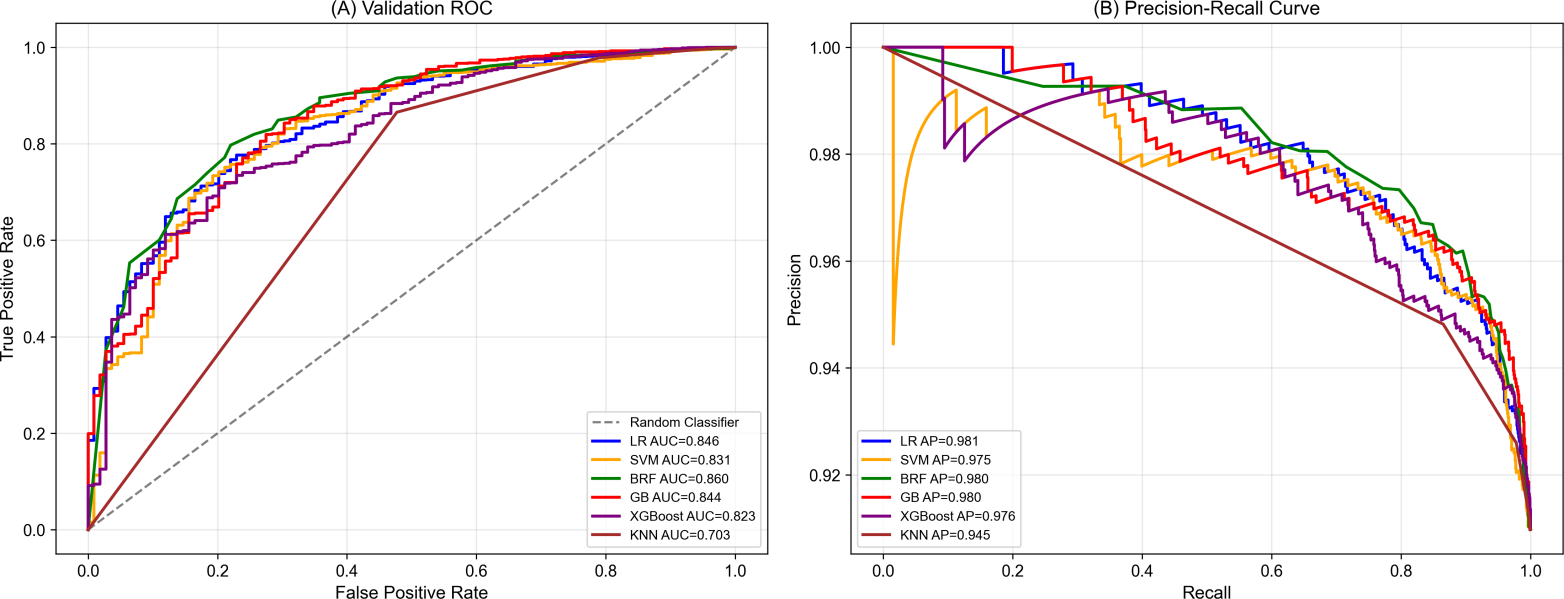
Performance comparison of 6 machine learning models. (A) ROC curves showing that LR and gradient boosting achieved the highest AUC (0.840), followed closely by random forest (0.836). (B) Precision-recall curves. Based on the balance between accuracy (0.856) and sensitivity (0.873) shown in Table 4, the BRF model was selected for the subsequent interpretability analysis. AP: average precision; AUC: area under the receiver operator characteristic curve; BRF: balanced random forest; GBDT: gradient boosting decision tree machine; KNN: *k*-nearest neighbor; LR: logistic regression; ML: machine learning; ROC: receiver operating characteristic; SVM: support vector machine; XGBoost: extreme gradient boosting.

Given the class imbalance in the dataset, the decision thresholds for each model were optimized according to the Youden index to achieve a balance between sensitivity and specificity [29]. Figure S1 in Multimedia Appendix 2 shows the results of threshold tuning analysis. As shown in Table 4, the LR model (AUC 0.840) and GB model (AUC 0.840) demonstrated superior predictive performance for physicians’ acceptance of AI medical tools compared with other ML models. Unadjusted (default) threshold results for all models are provided in Table S3 in Multimedia Appendix 1. Figure 5 shows the receiver operating characteristic and precision or recall curves for the 6 evaluated models. LR achieved the highest specificity (0.877) and average precision, whereas RF yielded the highest accuracy (0.856) and sensitivity (0.873).

**Table 4.** Comparison of various machine learning models’ performance.

Model	AUC^a^	Accuracy	Sensitivity	Specificity	*F*_1_-score	Youden index
RF^b^	0.836	0.856	0.873	0.685	0.917	0.558
LR^c^	0.840	0.682	0.663	0.877	0.791	0.539
GB^d^	0.840	0.794	0.799	0.740	0.876	0.539
XGBoost^e^	0.822	0.752	0.749	0.781	0.846	0.529
SVM^f^	0.806	0.745	0.742	0.781	0.841	0.523
KNN^g^	0.685	0.835	0.870	0.479	0.905	0.350

a AUC: area under the receiver operating characteristic curve.

b RF: random forest.

c LR: logistic regression.

d GB: gradient boosting.

e XGBoost: extreme gradient boosting.

f SVM: support vector machine.

g KNN: *k*-nearest neighbor.

Among all tested machine learning models, GB and LR achieved the highest performance with AUC of 0.840, followed by RF (AUC=0.836) and extreme gradient boosting (AUC=0.822). The detailed performance metrics for all models under unadjusted (default) thresholds are provided in Table S3 in Multimedia Appendix 1.

**Figure 6.**
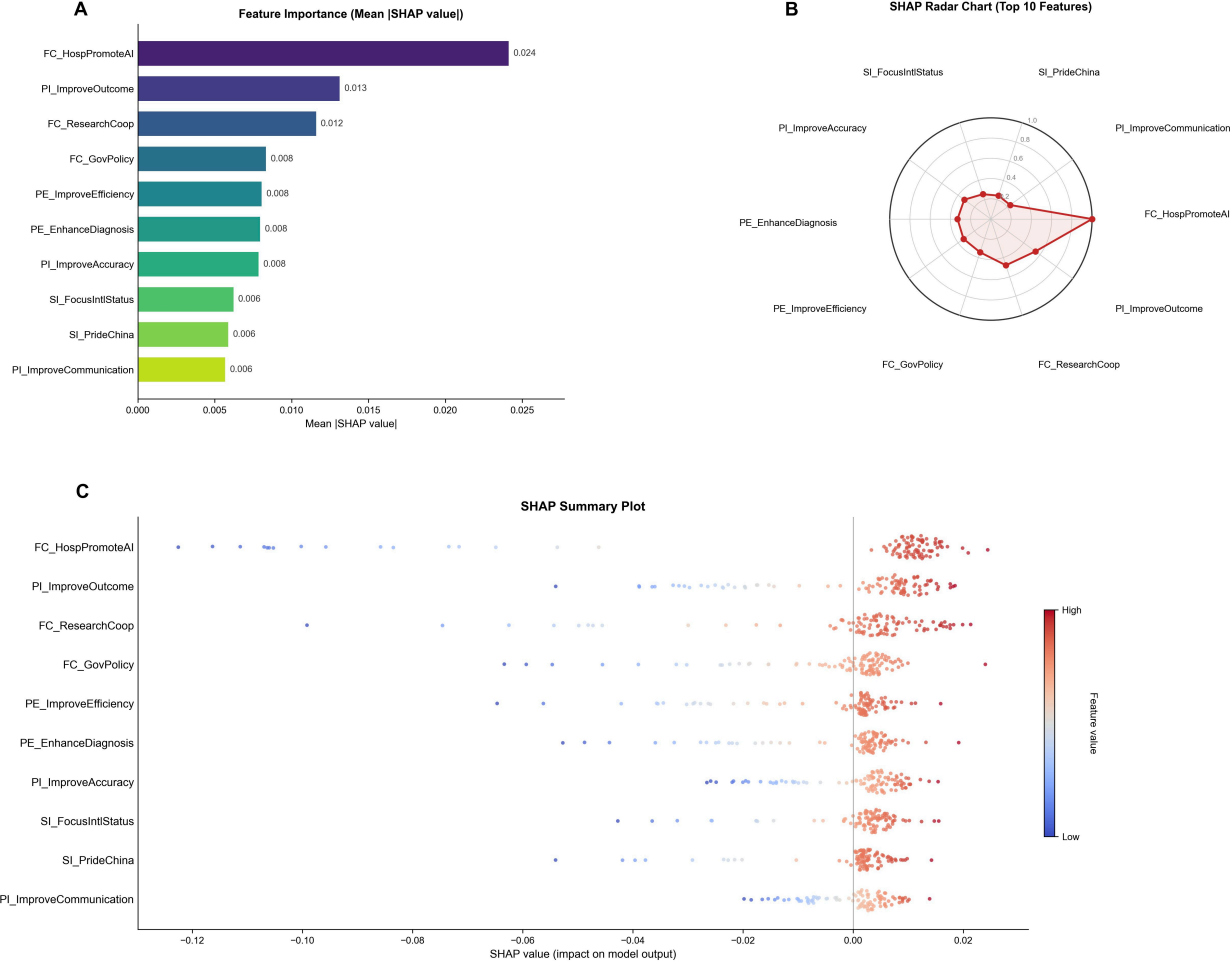
SHAP-based interpretability analysis of the optimal prediction model. (A) Mean absolute SHAP values ranking the top 10 most influential features for predicting physicians’ acceptance of artificial intelligence (AI) medical tools. (B) SHAP radar chart illustrating the relative importance of the top 10 features. (C) SHAP summary plot showing the distribution and direction of feature impacts. Higher perceived organizational support and clinical benefits of AI (red points) were associated with an increased probability of high acceptance, whereas lower scores (blue points) decreased the predicted acceptance. FC_HospPromoteAI: Hospitals should actively promote the use of AI medical tools; PI_ImproveOutcome: AI can help improve patients’ clinical outcomes; FC_ResearchCoop: Research institutions should strengthen AI-related research and collaboration; FC_GovPolicy: Government should introduce policies to promote AI in health care; PE_ImproveEfficiency: AI medical tools can improve health care efficiency; PE_EnhanceDiagnosis: AI medical tools can enhance physicians’ diagnostic ability; PI_ImproveAccuracy: AI can improve diagnostic accuracy; SI_FocusIntlStatus: I pay attention to China’s international status in medical AI; SI_PrideChina: I am proud of China’s rapid development in medical AI; PI_ImproveCommunication: AI can help improve communication between physicians and patients. FC: facilitating conditions; PE: performance expectancy; PI: positive impact; SHAP: Shapley Additive Explanations; SI: social influence.

The SHAP algorithm was applied to interpret the prediction of high acceptance of AI medical tools among physicians. Figure 6A shows the feature importance ranking with the top 10 predictors. FC_HospPromoteAI had the highest mean absolute SHAP value (0.024), though all effect sizes were modest, consistent with the ceiling effect in the outcome variable. Figure 6B presents the radar chart illustrating the relative importance of FC_HospPromoteAI compared to other variables. Figure 6C shows the SHAP summary plot, indicating that higher scores in organizational support (FC_HospPromoteAI) and perceived clinical benefits increased the predicted probability of high acceptance, while lower scores reduced it.

Decision curve analysis indicated that the GB classifier provided greater net benefit than both treat-all and treat-none strategies across most clinically plausible threshold probabilities (approximately 0.40 to 0.95; Figure S2 in Multimedia Appendix 2). In practice, this suggests that the model can help exploratorily identify characteristics associated with lower acceptance intentions, providing preliminary insights for targeted intervention design rather than definitive screening criteria. For individuals with these characteristics, administrators may consider initiating targeted interventions—specifically, reinforcing organizational support and demonstrating clinical evidence (as highlighted by SHAP)—rather than applying a one-size-fits-all training approach.

## Discussion

### Principal Findings

This study examined Chinese physicians’ acceptance of AI medical tools using an extended UTAUT framework combined with explainable machine learning. Acceptance was uniformly high (>90%) across subgroups, revealing a pronounced ceiling effect. In the structural model, PE, SI, FC, and PI were positive predictors of BI, whereas EE was not significant. Six negative moderation effects indicated that higher hospital level, greater AI familiarity, and stronger future optimism attenuated several main effects. In predictive analyses, the RF provided the best overall trade-off after threshold optimization, while LR and GB attained the highest AUC. SHAP interpretation ranked organizational support (“hospitals should actively promote AI”) and perceived clinical benefits (“AI improves outcomes” and “AI enhances diagnosis”) as the most influential features driving high acceptance.

While several path coefficients in the structural model were statistically significant yet relatively small in magnitude (PE: *β*=.03; SI: *β*=.02; PI: *β*=.01), these effects merit careful interpretation in context. First, the strong ceiling effect in BI (>90% acceptance across all dimensions) naturally constrains the range of variance available for prediction, mathematically limiting potential effect sizes. Second, in the presence of strong FC (*β*=.10, the largest predictor), the incremental contributions of PE and SI may be attenuated, as physicians already possess sufficient organizational support and resources to adopt AI regardless of individual perceptions. Third, from a practical significance perspective, even small standardized coefficients can translate to meaningful behavioral differences at population scale: a 0.03 increase in acceptance probability across China’s 4 million physicians represents over 100,000 additional potential AI adopters. Thus, while the effect sizes appear modest statistically, they reflect substantively important patterns within a highly accepting physician population operating under strong institutional support.

The strong ceiling effect in BI merits discussion. Rather than merely a psychometric limitation, this pattern reflects a substantive finding: Chinese physicians demonstrate remarkably high acceptance of medical AI technology across diverse applications (automated medical record generation, chronic disease management, diagnostic assistance, personalized treatment planning, medical imaging analysis, and surgical assistance). This high acceptance rate aligns with China’s national strategy to integrate AI into health care systems and suggests favorable conditions for medical AI implementation in Chinese health care settings.

Over 90% of physicians are willing to accept AI medical tools. Such a ceiling effect—wherein nearly all respondents express positive intent—demands careful interpretation. While high acceptance could reflect genuine enthusiasm driven by clinical needs and prior exposure to digital technologies, it may also result from social desirability bias or limitations in the measurement scale that restrict variance [30]. In contrast, studies from North America and Europe often report moderate acceptance levels. Recent multicountry surveys indicate that only 40%‐60% of physicians express readiness to integrate AI into routine practice [31,32]. Factors underlying this gap likely extend beyond technological maturity. In China, structural pressures—such as high patient volumes, long working hours, and relatively constrained staffing—may foster a pragmatic openness toward tools that promise efficiency gains. Government policy has also played a pivotal role [33]. However, a persistent challenge in behavioral research is the “attitude–behavior gap” [30]. High stated willingness does not guarantee sustained or effective use in practice. For example, follow-up studies in the United Kingdom found that despite favorable attitudes toward AI-based diagnostic support, actual use rates remained low due to workflow incompatibility and medico-legal concerns [34]. Thus, while this study’s findings provide optimism, they also highlight the need for longitudinal monitoring to assess whether intentions translate into ongoing adoption.

Within the extended UTAUT framework, 4 predictors—PE, SI, FC, and PI—emerged as statistically significant, whereas EE did not. *PE* consistently ranks as a central driver in technology adoption models. Physicians in our sample may perceive AI as capable of enhancing diagnostic accuracy, reducing manual workload, and improving patient outcomes, aligning with global findings that perceived clinical benefit is the most influential determinant of adoption [33,35]. This is particularly relevant in specialties, such as radiology and pathology, where AI applications have already demonstrated time-saving and accuracy-enhancing capabilities. *SI* reflects the role of peer networks, professional hierarchies, and institutional culture. In the Chinese health care system, hierarchical structures and strong alignment with administrative directives may amplify the influence of respected colleagues or hospital leadership [32]. In environments where senior physicians endorse AI tools, junior colleagues may be more inclined to adopt, regardless of personal reservations. FC captured organizational readiness, including training, IT infrastructure, and institutional support. The high predictive value of FC in this study aligns with recent organizational readiness research, which identifies supportive environments as critical enablers of AI deployment [36,37]. In resource-constrained settings, the absence of infrastructure and technical assistance often outweighs individual willingness as a barrier. The inclusion of *PI* as an extended UTAUT construct reflects an emerging recognition that clinicians also evaluate the societal and ethical implications of technology. Perceptions that AI could enhance health care equity, reduce medical errors, or improve doctor-patient relationships may bolster acceptance, particularly in systems under strain [38]. This dimension resonates with a broader shift in digital health evaluation frameworks toward sociotechnical models. The nonsignificant role of EE is noteworthy. In classical technology acceptance model and UTAUT studies, ease of use often exerts a strong influence on BI. Its absence here may signal several possibilities. First, the usability of current AI medical tools may have reached a level where interface complexity is no longer a major barrier for physicians already accustomed to electronic medical records. Second, the survey population consisted largely of physicians in tertiary hospitals (3115/4024, 77.4%), who may have greater access to IT support, thus minimizing perceived effort barriers [39].

The moderation analysis revealed 6 significant negative interaction effects, indicating that certain background factors attenuated the influence of main predictors. To better illustrate these patterns, simple slope analyses were conducted for the key moderators (Figure S3 in Multimedia Appendix 2). The results suggest a “substitution effect” mechanism. Specifically, higher hospital levels and greater AI familiarity reduced the impact of FC and SI on BI. Physicians in high-level hospitals or those familiar with AI likely possess sufficient internal resources and knowledge, making them less dependent on external organizational support or peer norms to form adoption intentions. In contrast, physicians with low familiarity (as shown by the steeper slopes in Figure S3A in Multimedia Appendix 2) rely heavily on external FC to bridge the gap in their own capability. Similarly, future optimism dampened the effects of PE. As shown in Figure S3B in Multimedia Appendix 2, physicians with high optimism maintain high adoption intentions regardless of the immediate performance gains, suggesting their internal belief acts as a buffer. Conversely, physicians with low optimism are more pragmatic; their willingness to adopt is highly sensitive to the tangible PE of the tools. This contrasts with the “wait-and-see” stance observed in previous studies, where anticipation of superior next-generation systems delays current uptake [40]. Instead of delaying adoption, our data suggests that high optimism provides a strong internal drive that substitutes for the need for immediate performance guarantees.

#### Methodological Synergy: Integration of SEM and Machine Learning

This study integrates SEM and ML to provide complementary perspectives on AI adoption. The SEM analysis established that FC (*β*=.10) was the strongest UTAUT predictor of BI. Correspondingly, SHAP analysis identified the item-level feature FC_HospPromoteAI (hospital organizational promotion of AI) as the most influential predictor of high acceptance (mean |SHAP|=0.024). This convergence between latent construct effects and item-level importance provides actionable specificity: interventions should prioritize institutional promotion and leadership endorsement rather than diffuse FC. Furthermore, SHAP revealed that physicians in secondary hospitals with moderate AI familiarity may respond differently to organizational support compared to tertiary hospital counterparts, suggesting the value of subgroup-specific implementation strategies. The complementary use of theory-driven SEM and data-driven ML thus validates the extended UTAUT framework while identifying concrete targets for management intervention.

#### Policy Implications: Evidence-Based Ethics and Governance

Our empirical findings suggest 3 policy priorities directly informed by the data. First, the dominance of FC_HospPromoteAI in SHAP analysis indicates that institutional endorsement is more influential than individual-level factors, supporting policies that mandate hospital-level AI governance committees and leadership commitment before deployment. Second, the negative moderation effect of AI familiarity on FC→BI (*β*=−.024) suggests that as physicians gain experience, their reliance on organizational support diminishes. This pattern has governance implications: early-stage implementation requires strong institutional backing and training, while mature adoption may benefit from peer-driven knowledge sharing and reduced top-down mandates. Third, the consistently high acceptance (>90%) but modest effect sizes suggest that the primary challenge is not physician willingness but rather structural readiness—policies should therefore prioritize infrastructure investment, workflow integration, and liability clarification over persuasion campaigns. Legal frameworks must clarify that AI serves as a decision support tool, with final clinical responsibility remaining with the physician.

### Limitations

This study has several limitations. First, while our sample achieved broad geographic coverage (29 of 31 provincial-level regions), it was skewed toward tertiary hospitals (77.4% vs 52.1% nationally) and physicians with senior titles (72.9% vs approximately 35% nationally). This distribution reflects two factors: (1) the user composition of the Medlive platform, which predominantly serves physicians in larger academic medical centers and (2) inherent regional differences in physician density across China. While we considered weighting adjustments, we opted to retain the raw data to avoid statistical instability from heavily weighting the smaller primary care subsample. These patterns may limit generalizability to primary care settings, junior physicians, and underrepresented regions. Future studies should use targeted recruitment strategies to ensure adequate representation of community health centers and rural clinics, where AI implementation may face distinct resource and infrastructure challenges.

Second, the AVE for the BI construct was 0.40, falling below the conventional 0.50 threshold. This reflects the variance constraint imposed by the dichotomous nature of the items and the pronounced ceiling effect (over 90% acceptance) observed in the data. However, the construct demonstrated high internal consistency (CR=0.80; Cronbach *α*=0.80). Furthermore, sensitivity analyses comparing factor loadings between ML and diagonally weighted least squares estimators showed minimal differences (mean loading difference=0.027, max=0.048; Table S4 in Multimedia Appendix 1), indicating that the choice of estimator did not substantively affect parameter estimates and the measurement model is robust.

Third, the generic framing of “AI medical tools” may overlook differences between specific application types. Finally, while explainable machine learning added predictive depth, it was not applied to longitudinal adoption data, which could further refine implementation strategies.

### Conclusion

Chinese physicians report an exceptionally high willingness to adopt AI medical tools, with organizational facilitation emerging as a key driver. Combining behavioral modeling with explainable ML highlights the relative importance of institutional readiness, though effect sizes are modest due to the pronounced ceiling effect. This underscores the need for context-specific strategies to translate intention into sustained, safe, and effective use.

## Supplementary material

10.2196/85270 Multimedia Appendix 1 Survey instrument items based on the extended Unified Theory of Acceptance and Use of Technology constructs and sociodemographic variables.

10.2196/85270 Multimedia Appendix 2 Threshold optimization for 6 classifiers and decision curve analysis for the primary model.

## Abbreviations

AI: artificial intelligence
AUC: area under the receiver operating characteristic curve
AVE: average variance extracted
BI: behavioral intention
CFI: comparative fit index
CR: composite reliability
EE: effort expectancy
FC: facilitating conditions
GB: gradient boosting
LR: logistic regression
ML: maximum likelihood
PE: performance expectancy
PE: performance expectancy
PI: positive impact
RF: random forest
RMSEA: root mean square error of approximation
SEM: structural equation modeling
SI: social influence
TLI: Tucker-Lewis index
UTAUT: Unified Theory of Acceptance and Use of Technology
