# Supplementary material for: Acceptance of Artificial Intelligence in Clinical Practice Among Chinese Physicians: Nationwide Cross-Sectional Survey Using Extended Unified Theory of Acceptance and Use of Technology and Explainable Machine Learning
Source: JMIR Med Inform. 2026 Apr 16;14:e85270. doi: 10.2196/85270 (PMC13086261; doi:10.2196/85270)
Supplement: Multimedia Appendix 1 [file medinform-v14-e85270-s001.docx]

**Multimedia Appendix 1. Supplementary Tables**

Table S1. Survey instrument items based on the extended UTAUT constructs and sociodemographic variables

**Background variables**

| Code | Question | Response options |
| --- | --- | --- |
| W5 | Hospital level | Primary / Secondary / Tertiary / Private / Other |
| W7 | Professional title | Junior / Intermediate / Associate senior / Chief senior / Ungraded |
| N1 | How familiar are you with AI technologies and related tools? | Heard of but unfamiliar / Completely unfamiliar / Somewhat familiar / Very familiar |
| N5 | When do you think AI will be widely adopted in healthcare? | Within the next year / 2–5 years / 5–10 years / >10 years / Will not be adopted / Hard to judge |
| L1 | In 2024, how did your overall income change? | +>51% / +31–50% / +10–30% / ~No change / −10–30% / −31–50% / −>51% |
| L2 | In 2024, how did your workload (outpatient, surgeries, etc.) change? | +>51% / +31–50% / +10–30% / ~No change / −10–30% / −31–50% / −>51% |

**Effort Expectancy (EE)**

| Code | Item | Scale |
| --- | --- | --- |
| N3_1 | I feel that using AI medical tools will increase my learning burden. | Likert (4-point) |
| N3_2 | I worry that I lack the ability to master AI tools. | Likert (4-point) |
| N3_3 | I find it difficult to understand how AI tools work. | Likert (4-point) |
| N3_4 | AI tools involve complex steps and are not convenient to use. | Likert (4-point) |
| N3_5 | It is hard for me to find time at work to learn AI tools. | Likert (4-point) |
| N3_6 | Repeated practice is needed before I can use AI tools proficiently. | Likert (4-point) |

**Performance Expectancy (PE)**

| Code | Item | Scale |
| --- | --- | --- |
| N6_1 | Promoting AI tools will increase physicians’ learning burden. | 4-point Likert |
| N6_2 | AI medical tools can improve healthcare efficiency. | 4-point Likert |
| N6_3 | AI medical tools can help reduce healthcare costs. | 4-point Likert |
| N6_4 | AI medical tools can enhance physicians’ diagnostic ability. | 4-point Likert |
| N6_5 | AI medical tools can improve patients’ care experience. | 4-point Likert |

**Perceived Positive Impact (PI)**

| **Code** | **Item** | **Scale** |
| --- | --- | --- |
| **N6_6** | **AI can help improve physician–patient communication.** | **4-point Likert** |
| **N6_7** | **AI can improve diagnostic accuracy.** | **4-point Likert** |
| **N6_8** | **AI can enhance patient safety and reduce misdiagnosis.** | **4-point Likert** |
| **N6_9** | **AI can help improve patients’ clinical outcomes.** | **4-point Likert** |

**Social Influence (SI)**

| Code | Item | Scale |
| --- | --- | --- |
| N7_1 | I am proud of China’s rapid development in medical AI. | 4-point Likert |
| N7_2 | I am willing to contribute to advancing medical AI in China. | 4-point Likert |
| N7_3 | China has a responsibility to lead global medical AI development. | 4-point Likert |
| N7_4 | I pay attention to China’s international standing in medical AI. | 4-point Likert |

**Facilitating Conditions (FC)**

| Code | Item | Scale |
| --- | --- | --- |
| N15_1 | Hospitals should actively promote the use of AI medical tools. | 4-point Likert |
| N15_2 | Government should introduce policies to promote AI in healthcare. | 4-point Likert |
| N15_3 | Research institutions should strengthen AI-related research and collaboration. | 4-point Likert |
| N15_4 | The development of medical AI requires multidisciplinary participation. | 4-point Likert |

**Behavioral Intention (BI)**

| Code | Item | Scale |
| --- | --- | --- |
| N10_1 | I am willing to learn to use AI-generated medical documentation tools. | Yes/No |
| N10_2 | I am willing to use AI-assisted chronic disease management tools. | Yes/No |
| N10_3 | I am willing to use AI-assisted diagnostic tools. | Yes/No |
| N10_4 | I am willing to use AI for personalized treatment planning. | Yes/No |
| N10_5 | I am willing to use AI for medical imaging diagnosis. | Yes/No |
| N10_6 | I am willing to use AI to assist surgical operations. | Yes/No |

*Note:* UTAUT, Unified Theory of Acceptance and Use of Technology; AI, artificial intelligence; BI, behavioral intention; EE, effort expectancy; PE, performance expectancy; PI, perceived positive impact; SI, social influence; FC, facilitating conditions.

Table S2. Factor loading matrix for all items (EFA)

| code | PE | BI | SI | FC | EE | PI |
| --- | --- | --- | --- | --- | --- | --- |
| N3_1 | 0.659 | 0.090 | 0.054 | 0.014 | 0.107 | 0.022 |
| N3_2 | 0.764 | 0.033 | 0.065 | 0.050 | 0.082 | 0.010 |
| N3_3 | 0.800 | 0.048 | 0.076 | 0.066 | 0.104 | 0.010 |
| N3_4 | 0.815 | 0.038 | 0.041 | 0.032 | 0.094 | 0.031 |
| N3_5 | 0.757 | 0.039 | 0.058 | 0.043 | 0.068 | 0.011 |
| N3_6 | 0.733 | 0.042 | 0.061 | 0.005 | 0.063 | 0.045 |
| N6_2 | 0.090 | 0.146 | 0.217 | 0.231 | 0.608 | 0.114 |
| N6_3 | 0.159 | 0.108 | 0.201 | 0.150 | 0.641 | 0.020 |
| N6_4 | 0.146 | 0.129 | 0.200 | 0.196 | 0.695 | 0.072 |
| N6_5 | 0.165 | 0.114 | 0.210 | 0.144 | 0.656 | 0.089 |
| N6_6 | 0.010 | 0.041 | 0.047 | 0.029 | 0.036 | 0.634 |
| N6_7 | 0.014 | 0.052 | 0.037 | 0.052 | 0.077 | 0.771 |
| N6_8 | 0.001 | 0.036 | 0.004 | 0.046 | 0.060 | 0.688 |
| N6_9 | 0.036 | 0.107 | 0.026 | 0.079 | 0.128 | 0.668 |
| N7_1 | 0.061 | 0.129 | 0.755 | 0.145 | 0.198 | 0.038 |
| N7_2 | 0.080 | 0.161 | 0.684 | 0.202 | 0.260 | 0.093 |
| N7_3 | 0.094 | 0.071 | 0.728 | 0.116 | 0.149 | 0.003 |
| N7_4 | 0.099 | 0.106 | 0.621 | 0.167 | 0.171 | 0.026 |
| N15_1 | 0.093 | 0.291 | 0.258 | 0.563 | 0.292 | 0.149 |
| N15_2 | 0.040 | 0.133 | 0.126 | 0.591 | 0.162 | 0.011 |
| N15_3 | 0.047 | 0.170 | 0.185 | 0.804 | 0.168 | 0.087 |
| N15_4 | 0.039 | 0.156 | 0.174 | 0.769 | 0.169 | 0.094 |
| N10_1 | 0.044 | 0.559 | 0.097 | 0.136 | 0.120 | 0.038 |
| N10_2 | 0.038 | 0.572 | 0.122 | 0.061 | 0.007 | 0.017 |
| N10_3 | 0.029 | 0.704 | 0.061 | 0.099 | 0.061 | 0.042 |
| N10_4 | 0.078 | 0.569 | 0.005 | 0.104 | 0.084 | 0.073 |
| N10_5 | 0.017 | 0.639 | 0.057 | 0.048 | 0.057 | 0.039 |
| N10_6 | 0.049 | 0.599 | 0.071 | 0.092 | 0.104 | 0.054 |

Table S3. Model performance at default thresholds (unadjusted)

| Model | AUC | ACC | SEN | SPE | F1 | Youden |
| --- | --- | --- | --- | --- | --- | --- |
| RF | 0.836 | 0.913 | 0.993 | 0.110 | 0.954 | 0.836 |
| LR | 0.840 | 0.912 | 0.981 | 0.219 | 0.953 | 0.840 |
| GB | 0.840 | 0.911 | 0.995 | 0.068 | 0.953 | 0.539 |
| XGBoost | 0.822 | 0.902 | 0.971 | 0.205 | 0.947 | 0.822 |
| SVM | 0.806 | 0.867 | 0.899 | 0.548 | 0.925 | 0.806 |
| KNN | 0.685 | 0.804 | 0.817 | 0.671 | 0.883 | 0.330 |

Note: AUC, area under the ROC curve; ACC, accuracy; SEN, sensitivity; SPE, specificity; RF, random forest; LR, logistic regression; GB, gradient boosting; SVM, support vector machine; KNN, k-nearest neighbors.

Table S4. Comparison of Standardized Factor Loadings for BI Items: ML vs. DWLS Estimators

| Item | Ceiling Effect (%) | ML Loading | DWLS Loading | Difference |
| --- | --- | --- | --- | --- |
| N10_1 | 92.6 | 1.000 | 1.000 | 0.000 |
| N10_2 | 96.4 | 0.721 | 0.723 | 0.003 |
| N10_3 | 95.5 | 0.909 | 0.958 | 0.048 |
| N10_4 | 93.1 | 1.016 | 0.988 | 0.029 |
| N10_5 | 95.6 | 0.856 | 0.893 | 0.037 |
| N10_6 | 90.6 | 1.215 | 1.169 | 0.047 |
| Mean | 93.96 | - | - | 0.027 |

Note: All loadings are standardized relative to the first item (N10_1). The minimal differences indicate robust parameter estimation across estimators despite the strong ceiling effect in BI items.
