# Supplementary material for: Acceptance of Artificial Intelligence in Clinical Practice Among Chinese Physicians: Nationwide Cross-Sectional Survey Using Extended Unified Theory of Acceptance and Use of Technology and Explainable Machine Learning
Source: JMIR Med Inform. 2026 Apr 16;14:e85270. doi: 10.2196/85270 (PMC13086261; doi:10.2196/85270)
Supplement: Multimedia Appendix 2 [file medinform-v14-e85270-s002.docx]

**Multimedia Appendix 2. Supplementary Figures**

| 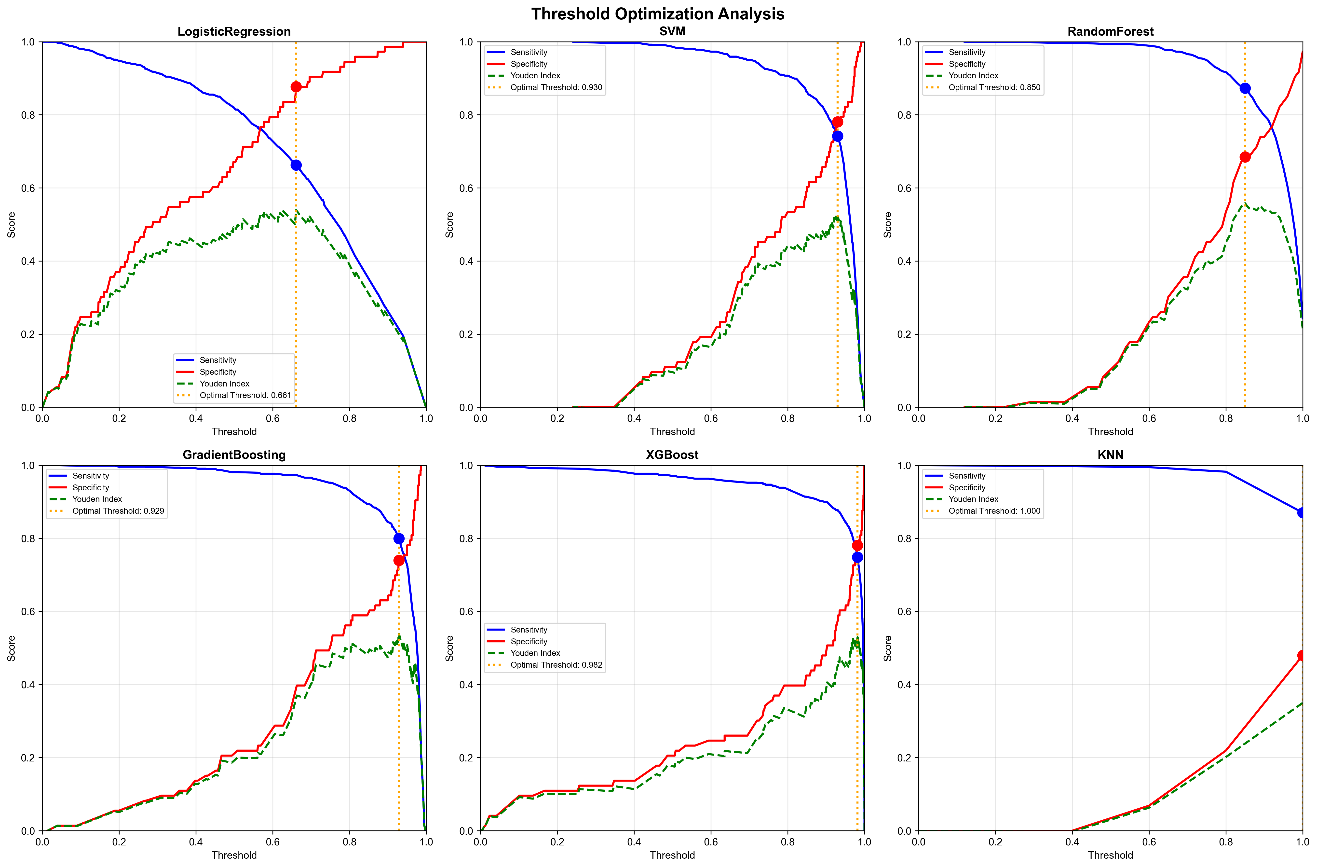Figure S1. Threshold optimization for six classifiers.  Curves show sensitivity (blue; true positive rate), specificity (red; true negative rate), and Youden’s J (green dashed; J = sensitivity + specificity − 1) across decision thresholds (x-axis). The vertical orange dotted line marks the optimal threshold that maximizes Youden’s J. The blue dot and red dot denote the sensitivity and specificity achieved at that optimal threshold, respectively. |
| --- |


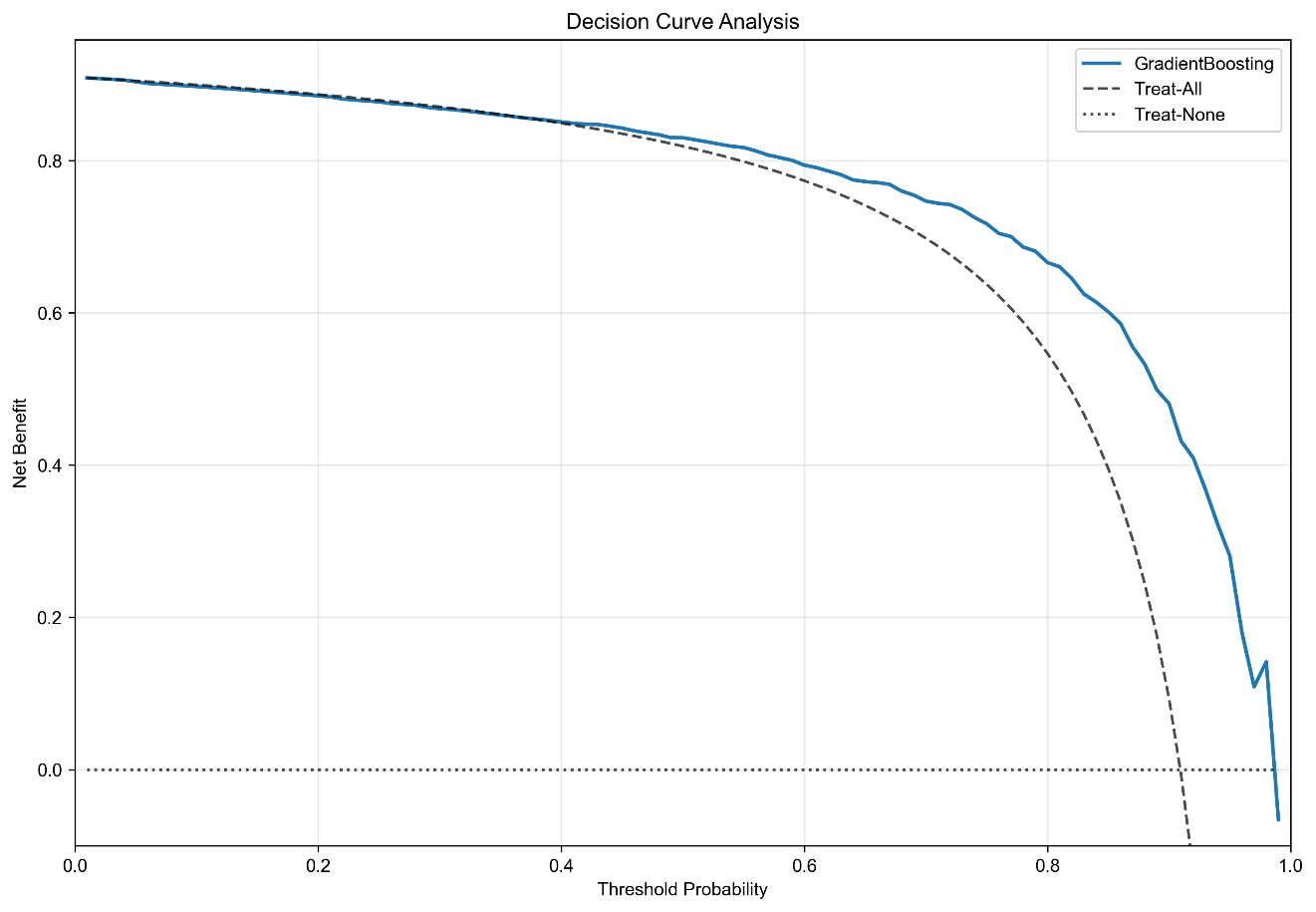
Figure S2. Decision curve analysis (DCA) for the primary model.
Net benefit across a range of threshold probabilities for the main classifier, compared with “treat-all” and “treat-none” strategies.


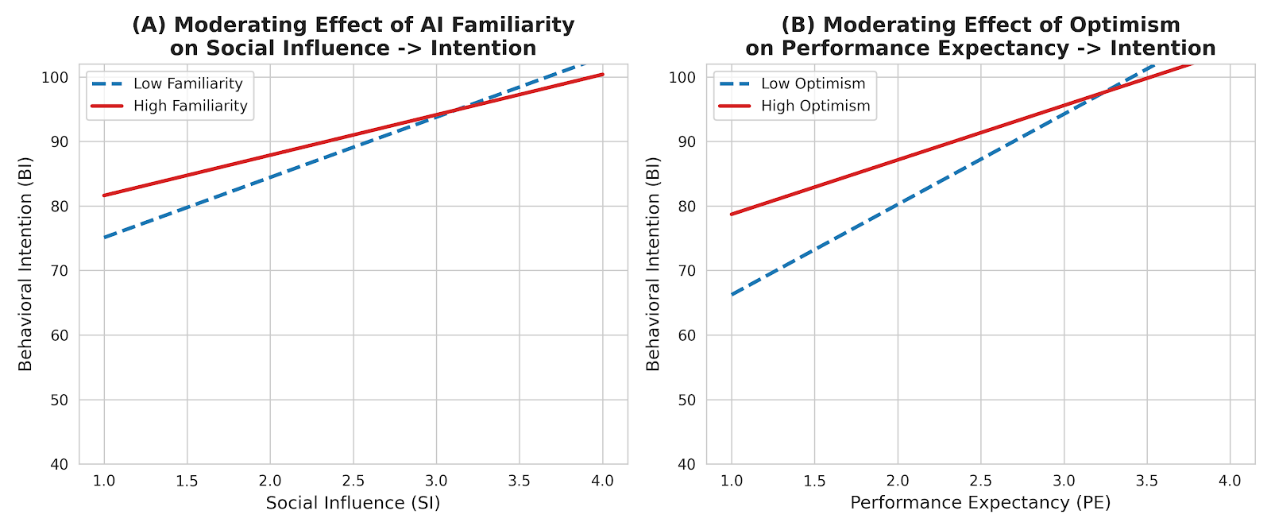


Figure S3. Simple slope analyses illustrating the negative moderation effects. (A) The moderating effect of AI Familiarity on the relationship between Social Influence (SI) and Behavioral Intention (BI). The slope is steeper for physicians with low familiarity (dashed blue line), indicating they rely more heavily on social influence to form adoption intentions compared to those with high familiarity (solid red line). (B) The moderating effect of Optimism on the relationship between Performance Expectancy (PE) and Behavioral Intention (BI). Similarly, physicians with lower optimism (dashed blue line) show a stronger dependency on performance benefits, whereas highly optimistic physicians maintain high intention levels regardless of performance expectancy, demonstrating a "substitution effect."
